# The Impact of COVID-19 Lockdown on Cases of and Deaths From AIDS, Gonorrhea, Syphilis, Hepatitis B, and Hepatitis C: Interrupted Time Series Analysis

**DOI:** 10.2196/40591

**Published:** 2023-05-03

**Authors:** Xinsheng Wu, Xinyi Zhou, Yuanyi Chen, Ke Zhai, Ruoyao Sun, Ganfeng Luo, Yi-Fan Lin, Yuwei Li, Chongguang Yang, Huachun Zou

**Affiliations:** 1 School of Public Health (Shenzhen) Sun Yat-sen University Shenzhen, Guangdong China

**Keywords:** COVID-19, AIDS, gonorrhea, syphilis, hepatitis B, hepatitis C, China, nonpharmaceutical, intervention, STD, disease, virus, cases, deaths, fatality, data

## Abstract

**Background:**

China implemented a nationwide lockdown to contain COVID-19 from an early stage. Previous studies of the impact of COVID-19 on sexually transmitted diseases (STDs) and diseases caused by blood-borne viruses (BBVs) in China have yielded widely disparate results, and studies on deaths attributable to STDs and BBVs are scarce.

**Objective:**

We aimed to elucidate the impact of COVID-19 lockdown on cases, deaths, and case-fatality ratios of STDs and BBVs.

**Methods:**

We extracted monthly data on cases and deaths for AIDS, gonorrhea, syphilis, hepatitis B, and hepatitis C between January 2015 and December 2021 from the notifiable disease reporting database on the official website of the National Health Commission of China. We used descriptive statistics to summarize the number of cases and deaths and calculated incidence and case-fatality ratios before and after the implementation of a nationwide lockdown (in January 2020). We used negative binominal segmented regression models to estimate the immediate and long-term impacts of lockdown on cases, deaths, and case-fatality ratios in January 2020 and December 2021, respectively.

**Results:**

A total of 14,800,330 cases of and 127,030 deaths from AIDS, gonorrhea, syphilis, hepatitis B, and hepatitis C were reported from January 2015 to December 2021, with an incidence of 149.11/100,000 before lockdown and 151.41/100,000 after lockdown and a case-fatality ratio of 8.21/1000 before lockdown and 9.50/1000 after lockdown. The negative binominal model showed significant decreases in January 2020 in AIDS cases (–23.4%; incidence rate ratio [IRR] 0.766, 95% CI 0.626-0.939) and deaths (–23.9%; IRR 0.761, 95% CI 0.647-0.896), gonorrhea cases (–34.3%; IRR 0.657, 95% CI 0.524-0.823), syphilis cases (–15.4%; IRR 0.846, 95% CI 0.763-0.937), hepatitis B cases (–17.5%; IRR 0.825, 95% CI 0.726-0.937), and hepatitis C cases (–19.6%; IRR 0.804, 95% CI 0.693-0.933). Gonorrhea, syphilis, and hepatitis C showed small increases in the number of deaths and case-fatality ratios in January 2020. By December 2021, the cases, deaths, and case-fatality ratios for each disease had either reached or remained below expected levels.

**Conclusions:**

COVID-19 lockdown may have contributed to fewer reported cases of AIDS, gonorrhea, syphilis, hepatitis B, and hepatitis C and more reported deaths and case-fatality ratios of gonorrhea, syphilis, and hepatitis C in China.

## Introduction

China implemented stringent nonpharmaceutical interventions (NPIs), including city lockdowns and traffic restrictions, social distancing, and school closures in the early stages of the outbreak of COVID-19 to contain its spread [1-7]. These NPIs contributed to low morbidity and mortality of COVID-19 in the country [8-10]. However, it is necessary to keep a constant focus on other important infectious diseases. The numbers of documented influenza and pneumonia deaths in the United States in 2020 were reported to increase by 7.5% compared to 2019 [11], and modeling predicted that HIV deaths in African countries would increase [12,13].

As humans migrate and communicate, sexually transmitted diseases (STDs) and diseases caused by blood-borne viruses (BBVs) are a serious global burden [14-16]. Although China has established a national STD surveillance sentinel system, increased publicity and education, and improved treatment guidelines, STDs and BBVs are still on the rise. HIV/AIDS morbidity, mortality, and new HIV infections (defined as newly acquired blood HIV antibodies, as shown by positive results in ELISA and Western blotting and a large viral load in PCR testing) continually increased each year in China from 2004 to 2016 (from 0.235/100,000, 0.057/100, and 1.020/100,000 in 2004, respectively, to 3.990/100,000, 1.034/100, and 6.442/100,000, respectively, in 2016) [17]. Surveillance data from 2004 to 2013 in China showed that the incidence of hepatitis C, HIV infection, and syphilis increased by 19.2% (95% CI 15.9%-22.6%) annually [18].

Understanding changes in AIDS, gonorrhea, syphilis, hepatitis B, and hepatitis C over time is vital to contain the disease burdens of STDs and BBVs. Many studies have mainly focused on changes in respiratory infectious diseases during COVID-19 [19-21]. There is limited and conflicting evidence on changes in STDs and BBVs during the pandemic. A large tertiary care hospital in Chicago [22] reported the rate of presumed active infection with syphilis increased from 1.2% (prepandemic; June 2019-March 2020) to 1.8% (during the pandemic; April 2020-June 2020). Two referral centers in Greece [23] recorded 10% and 36.9% decreases in syphilis and gonorrhea cases, respectively, in 2020. A study of the STD and BBV service of an Italian clinic [24] did not find statistically significant differences compared with the previous 4 years. In addition, previous studies of the impact of COVID-19 on STDs and BBVs in China have yielded widely disparate results [25,26], and data on deaths attributable to STDs and BBVs are scarce. We aimed to elucidate the impact of COVID-19 lockdown on cases, deaths, and case-fatality ratios for AIDS, gonorrhea, syphilis, hepatitis B, and hepatitis C in China using interrupted time series analysis. This may provide evidence of the impact of COVID-19 lockdown on STDs and BBVs at the national level and inform planning for efficient control strategies in the postpandemic era.

## Methods

### Study Design

China launched a national online routine reporting system for selected infectious diseases in 2003 that covers all 31 provinces in mainland China. Health facilities at various levels are required to report cases through standard case report forms within 2 to 24 hours of the detection of a notifiable infectious disease.

We extracted national data on monthly reported cases and deaths for AIDS, gonorrhea, syphilis, hepatitis B, and hepatitis C, which are mainly or can be sexually transmitted, from January 2015 to December 2021 from the official website of the China Health and Wellness Commission [27]. The lockdown period was considered to start in January 2020 with the Wuhan city lockdown (on January 23, 2020) and massive restrictions implemented nationwide. Although some regions had eased restrictions before April 8 (when Wuhan lifted its restrictions), they tended to take stronger NPI measures to deal with the possible importation of COVID-19 cases from Wuhan. Based on these considerations, we defined the lockdown period as ending in April 2020. AIDS cases are defined as a diagnosis of AIDS. Syphilis cases include both primary and secondary syphilis. Cases that were reported as cases of notifiable infectious disease and died as a result of that infectious disease are reported as deaths, excluding deaths due to accidents or noninfectious diseases.

### Statistical Analysis

The primary outcome was the number of cases and deaths reported by month for AIDS, gonorrhea, syphilis, hepatitis B, and hepatitis C before and after lockdown implementation. The secondary outcomes were the incidence and case-fatality ratios. We defined the incidence (per 100,000 population) as the mean value of annual new reported cases divided by the population size and the case-fatality ratio (per 1000 population) as the mean value of annual reported deaths divided by annual new reported cases.

We first summarized monthly reported data on cases and deaths for the 5 STDs and BBVs as the median (IQR) before, during, and after lockdown and calculated the incidence and case-fatality ratios before and after lockdown. The Kruskal-Wallis test was used to compare secondary outcomes before and after lockdown. Since overdispersion occurred in almost all outcomes (Multimedia Appendix 1, Table S1), we conducted an interrupted time series analysis by fitting a negative binominal segmented regression model to estimate the immediate effects of lockdown on cases, deaths, and case-fatality ratios, as well as trends after lockdown [28]. The negative binominal model included (1) a time variable, (2) a dummy variable representing the pre- and postlockdown periods, and (3) an interaction term between time and the dummy variables (Multimedia Appendix 1).

We adjusted for seasonality and long-term trends to control their effects and allow the direct study of changes in public health issues (ie, testing and transmission), as follows: (1) the reporting of STD and BBV data is influenced by individual and clinical activity, so we adjusted for seasonal variation by including a Fourier term consisting of 2 sine-cosine pairs in the model [29]; (2) the reporting of STD and BBV data has long-term trends, so we adjusted for long-term trends by using the prelockdown model to predict the expected outcomes after lockdown (ie, a counterfactual scenario). We analyzed the annual trends of the models for the 5 diseases to determine their seasonality.

The incidence rate ratio (IRR) was calculated by comparing the fitted numbers from the model with the expected numbers from the contemporaneous counterfactual. By subtracting a period from the time variable, we were able to center time in January 2020 and December 2021 to estimate the impact on different timepoints [30]. To calculate the trend after lockdown, we added the coefficients associated with time and the time-dummy interaction. We adjusted the standard errors of the model parameters using the Newey-West method to calculate a 95% CI for the IRR in both January 2020 and December 2021, with lag taking the optimal value calculated [31].

We conducted a sensitivity analysis of the number of pairs of Fourier terms (1, 3, and 5 pairs). All statistical tests were 2-sided, and *P*<.05 was considered statistically significant. We performed all analyses in R (version 4.0.4; R Foundation for Statistical Computing; Multimedia Appendix 1).

### Ethical Considerations

This study was a secondary analysis of online reported data with no identifying information available to the researchers. Therefore, ethical review was not applicable.

## Results

### Overview of Study

**Figure 1:**
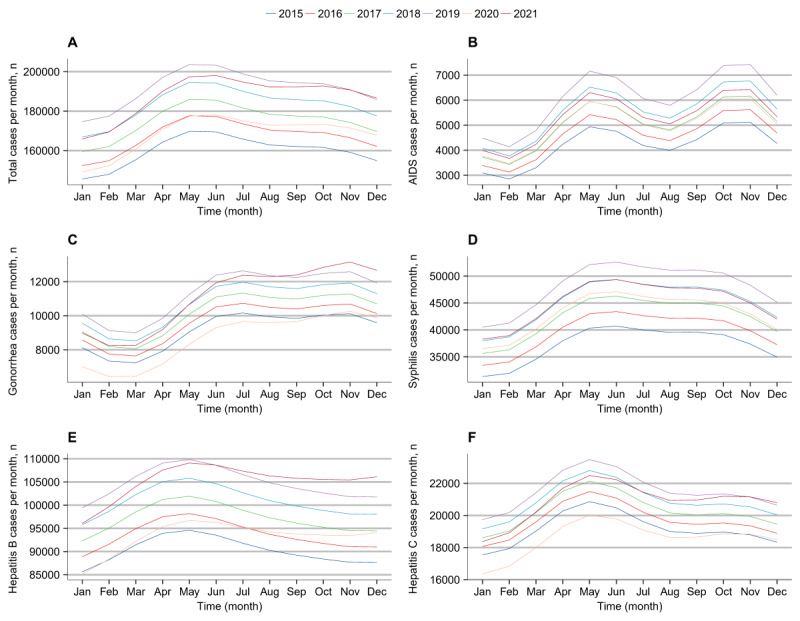
Negative binominal models for monthly cases of AIDS, gonorrhea, syphilis, hepatitis B, and hepatitis C by year in China from January 2015 to December 2021. Total cases (A), AIDS (B), gonorrhea (C), syphilis (D), hepatitis B (E) and hepatitis C (F).

From January 2015 to December 2021, 14,800,330 cases of AIDS, gonorrhea, syphilis, hepatitis B, and hepatitis C were reported in China, with a notified incidence of 149.11/100,000 before lockdown and 151.41/100,000 after lockdown. The incidence (mean value of annual incidence) change in these 5 infectious diseases before and after lockdown is shown in Table 1. There were slight but not significant (*P*>.05) changes in the incidence of the 5 diseases after lockdown. The median number of monthly reported cases was 177,556 (IQR 164,546-187,722) during the 60 months before lockdown, 155,491 (IQR 121,585-157,244) during the first 3 months of nationwide lockdown (January 2020 to March 2020), and 184,401 (IQR 180,396-190,466) during the 21 months after lockdown eased (April 2020 to December 2021). Figure 1 shows the annual seasonal pattern in the case counts of the 5 diseases. AIDS peaked in May and October to November, gonorrhea in July and November, syphilis in May to June, and hepatitis B and C in May.

In total, 127,030 deaths were reported during the study period, with the case-fatality ratio changing from 8.15/1000 before lockdown to 9.50/1000 after lockdown. The change in case-fatality ratios (ie, the mean value of annual case-fatality ratios) for the 5 infectious diseases before and after lockdown is shown in Table 2. There was a significant (*P*=.03) increase in the gonorrhea case-fatality ratio after lockdown (from 0.01/1000 to 0.04/1000). The median number of monthly deaths due to the five STDs and BBVs was 1360 (IQR 1208-1692) before lockdown, 1078 (IQR 1054-1270) during lockdown, and 1761 (IQR 1602-1942) after lockdown eased.

**Table 1:** Case numbers and incidence rates for AIDS, gonorrhea, syphilis, hepatitis B, and hepatitis C in China from January 2015 to December 2021. Incidence was calculated as the mean number of annual new cases divided by the population size (per 100,000).

	Cases^a^, n	Incidence before lockdown^b,c^	Incidence after lockdown^b,d^	*P* value	Cases before lockdown^c,e^, median (IQR)	Cases during lockdown^e,f^, median (IQR)	Cases after lockdown eased^e,g^, median (IQR)
All 5 diseases	14,800,330	149.11	151.41	.70	177,556 (164,546-187,722)	155,491 (121,585-157,244)	184,401 (180,396-190,466)
AIDS	428,715	4.31	4.40	.70	5124 (4291-6098)	2759 (2446-3784)	5484 (5039-6124)
Gonorrhea	846,935	8.65	8.38	.70	10,436 (8954-11,343)	4661 (4092-6458)	10,874 (10,551-11,264)
Syphilis	3,612,684	36.15	37.56	.44	42,702 (38,898-46,394)	39,671 (30,560-40,412)	46,538 (44,438-47,999)
Hepatitis B	8,219,292	82.72	84.29	.70	97,461 (90,780-103,851)	88,150 (69,828-89,588)	101,701 (99,319-105,393)
Hepatitis C	1,692,704	17.27	16.78	.44	20,400 (19,224-21,502)	16,718 (12,893-17,002)	20,438 (20,001-21,254)

^a^Total number of cases from January 2015 to December 2021.

^b^Per year.

^c^Before lockdown: January 2015 to December 2019.

^d^After lockdown: From January 2020 to December 2021.

^e^Per month.

^f^During lockdown: From January 2020 to March 2020.

^g^After lockdown eased: From April 2020 to December 2021.

**Table 2:** Deaths and case-fatality ratios for AIDS, gonorrhea, syphilis, hepatitis B, and hepatitis C in China from January 2015 to December 2021. The case-fatality ratio represents the mean number of annual deaths divided by annual new cases (per 1000).

	Deaths^a^, n	Case-fatality ratio before lockdown^b,c^	Case-fatality ratio after lockdown^c,d^	*P* value	Deaths before lockdown^b,e^, median (IQR)	Deaths during lockdown^e,f^, median (IQR)	Deaths after lockdown eased^e,g^, median (IQR)
All 5 diseases	127,030	8.15	9.50	.44	1360 (1208-1692)	1078 (1054-1270)	1761 (1602-1942)
AIDS	122,508	271.38	316.23	.44	1304 (1156-1635)	971 (962-1194)	1700 (1554-1874)
Gonorrhea	15	0.01	0.04	.03	0 (0-0)	0 (0-2)	0 (0-0)
Syphilis	516	0.14	0.16	>.99	6 (4-8)	9 (8-17)	6 (4-7)
Hepatitis B	3168	0.38	0.41	.24	38 (31-42)	37 (34-58)	37 (33-44)
Hepatitis C	823	0.49	0.48	.70	9 (7-12)	12 (9-14)	10 (8-11)

^a^Total number from January 2015 to December 2021.

^b^Before lockdown: From January 2015 to December 2019.

^c^Per year.

^d^After lockdown: From January 2020 to December 2021.

^e^Per month.

^f^During lockdown: From January 2020 to March 2020.

^g^After lockdown eased: From April 2020 to December 2021.

### Cases and Deaths

#### AIDS

**Figure 2:**
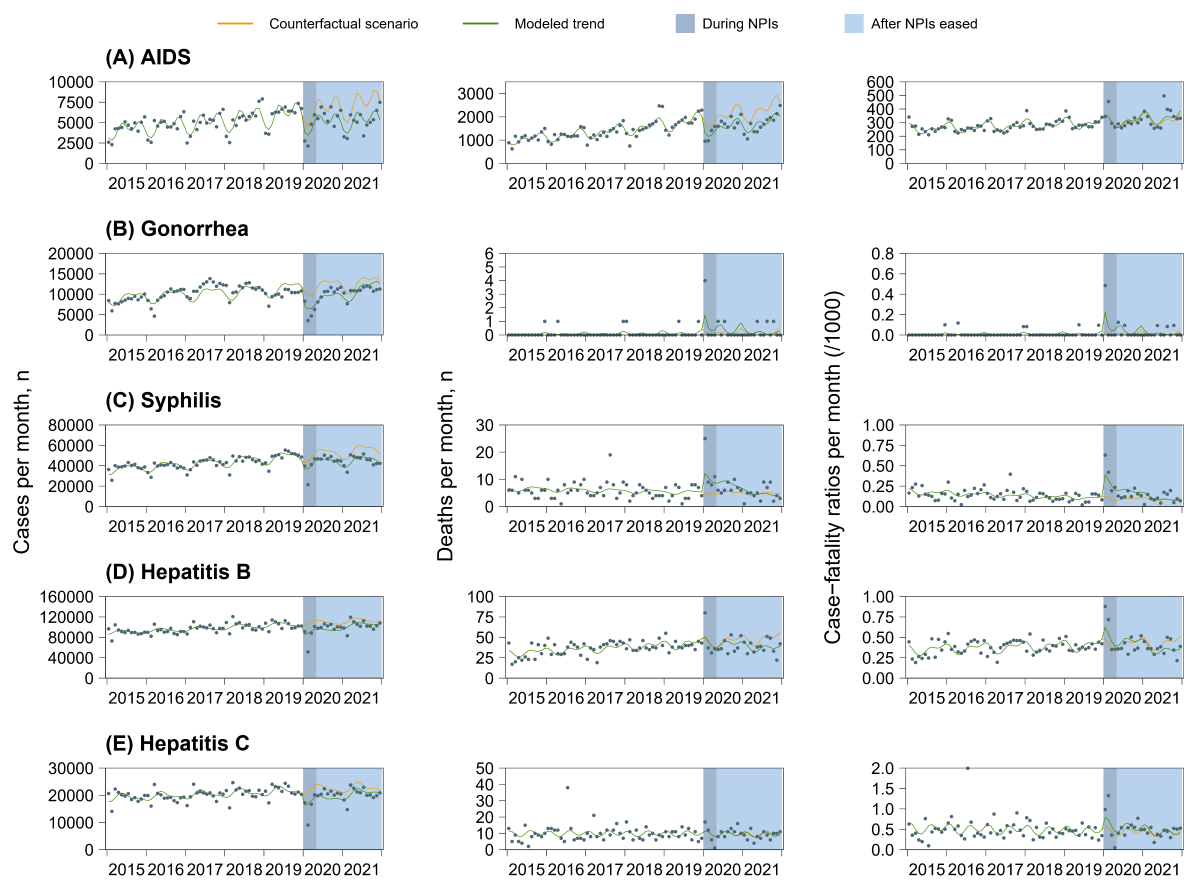
Monthly numbers, trends, and fitted negative binominal segmented regression models for 5 sexually transmitted diseases and diseases caused by blood-borne viruses in China from January 2015 to December 2021: AIDS (A), gonorrhea (B), syphilis (C), hepatitis B (D), and hepatitis C (E). The light blue shaded areas indicate the period during the lockdown, from January 2020 to March 2020, and the dark blue shaded areas indicate the period after lockdown eased, from April 2020 to December 2021. Case-fatality ratios represent the number of deaths divided by the number of new cases (per 1000). NPI: nonpharmaceutical intervention.

In the first month of lockdown, there was a 23.4% (IRR 0.766, 95% CI 0.626-0.939; Figure 2A and Table 3) decrease in the number of reported AIDS cases; after lockdown, the monthly number remained unchanged (IRR 1.005, 95% CI 0.990-1.020); by December 2021, AIDS cases were still below the expected level (IRR 0.715, 95% CI 0.586-0.874).

In the first month of lockdown, the number of reported AIDS deaths showed a 23.9% decline (IRR 0.761, 95% CI 0.647-0.896); after lockdown, the number showed an increasing trend of 1.1% (IRR 1.011, 95% CI 1.000-1.021) per month; by December 2021, the number of AIDS deaths remained below the expected level (IRR 0.754, 95% CI 0.654-0.869). In the first month of lockdown, there was no significant change in the AIDS case-fatality ratio (IRR 1.004, 95% CI 0.895-1.125); after lockdown, the monthly number remained unchanged (IRR 1.006, 95% CI 0.997-1.016); by December 2021, the AIDS case-fatality ratio remained in line with the expected level (IRR 1.075, 95% CI 0.924-1.252).

**Table 3:** Negative binominal segmented regression models of the impact of COVID-19 lockdown on AIDS, gonorrhea, syphilis, hepatitis B, and hepatitis C in China from January 2015 to December 2021. Trend represents the slope change per month. Case-fatality ratio represents the number of deaths divided by the number of new cases. Autocorrelation was addressed for all diseases using Newey-West standard errors to calculate CIs, with lag taking the optimal value calculated.

Disease		IRR^a^ (95% CI) at lockdown^b^	IRR (95% CI) at study end^c^	Trend (95% CI) before lockdown^d^	Trend (95% CI) after lockdown^e^
**AIDS**					
	Cases	0.766 (0.626-0.939)	0.715 (0.586-0.874)	1.008 (1.006-1.009)	1.005 (0.990-1.020)
	Deaths	0.761 (0.647-0.896)	0.754 (0.654-0.869)	1.011 (1.009-1.013)	1.011 (1.000-1.021)
	Case-fatality ratio	1.004 (0.895-1.125)	1.075 (0.924-1.252)	1.003 (1.002-1.004)	1.006 (0.997-1.016)
**Gonorrhea**					
	Cases	0.657 (0.524-0.823)	0.952 (0.722-1.257)	1.005 (1.001-1.008)	1.021 (1.007-1.035)
	Deaths	5.902 (1.090-31.959)	0.558 (0.038-8.266)	1.015 (0.982-1.049)	0.916 (0.804-1.043)
	Case-fatality ratio	9.676 (1.764-53.089)	0.398 (0.018-8.890)	1.012 (0.977-1.048)	0.880 (0.766-1.012)
**Syphilis**					
	Cases	0.846 (0.763-0.937)	0.818 (0.751-0.892)	1.005 (1.004-1.006)	1.004 (0.998-1.010)
	Deaths	2.380 (1.455-3.893)	0.735 (0.421-1.284)	0.995 (0.990-1.001)	0.946 (0.913-0.980)
	Case-fatality ratio	3.173 (1.877-5.365)	0.810 (0.442-1.484)	0.990 (0.984-0.996)	0.933 (0.897-0.971)
**Hepatitis B**					
	Cases	0.825 (0.726-0.937)	0.968 (0.896-1.045)	1.003 (1.002-1.004)	1.010 (1.002-1.018)
	Deaths	1.038 (0.854-1.261)	0.704 (0.590-0.842)	1.006 (1.003-1.009)	0.989 (0.979-0.999)
	Case-fatality ratio	1.312 (0.997-1.726)	0.709 (0.556-0.902)	1.003 (1.000-1.006)	0.976 (0.958-0.995)
**Hepatitis C**					
	Cases	0.804 (0.693-0.933)	0.950 (0.854-1.057)	1.002 (1.001-1.004)	1.010 (1.001-1.019)
	Deaths	1.006 (0.781-1.296)	0.926 (0.672-1.275)	1.000 (0.994-1.006)	0.996 (0.982-1.011)
	Case-fatality ratio	1.394 (1.080-1.800)	0.906 (0.653-1.258)	0.997 (0.991-1.004)	0.979 (0.966-0.992)

^a^IRR: incidence rate ratio.

^b^January 2020.

^c^December 2021.

^d^From January 2015 to December 2019.

^e^From January 2020 to December 2021.

#### Gonorrhea

In the first month of lockdown, there was a 34.3% decline (IRR 0.657, 95% CI 0.524-0.823; Figure 2B and Table 3) in the number of reported gonorrhea cases; after lockdown, there was a 2.1% (IRR 1.021, 95% CI 1.007-1.035) monthly increasing trend; by December 2021, the number of gonorrhea cases had returned to the expected level (IRR 0.952, 95% CI 0.722-1.257).

In the first month of lockdown, the number of reported gonorrhea deaths showed an increase of 490.2% (IRR 5.902, 95% CI 1.090-31.959); after lockdown, the number remained largely unchanged (IRR 0.916, 95% CI 0.804-1.043); by December 2021, the number of gonorrhea deaths had returned to below the expected level (IRR 0.558, 95% CI 0.038-8.266). In the first month of lockdown, there was an 867.6% increase (IRR 9.676, 95% CI 1.764-53.089) in the gonorrhea case-fatality ratio; after lockdown, the number remained unchanged (IRR 0.880, 95% CI 0.766-1.012); by August 2021, the gonorrhea case-fatality ratio had returned to below the expected level (IRR 0.398, 95% CI 0.018-8.890).

#### Syphilis

In the first month of lockdown, the number of reported syphilis cases showed a 15.4% decrease (IRR 0.846, 95% CI 0.763-0.937; Figure 2C and Table 3); after lockdown, the monthly number per month was unchanged (IRR 1.004, 95% CI 0.998-1.010); by December 2021, the number of syphilis cases was still below the expected level (IRR 0.818, 95% CI 0.751-0.892).

In the first month of lockdown, the number of reported syphilis deaths showed an increase of 138% (IRR 2.380, 95% CI 1.455-3.893); after lockdown, the number per month showed a decreasing trend of 5.4% (IRR 0.946, 95% CI 0.913-0.980); by December 2021, the number of syphilis deaths had returned to below the expected level (IRR 0.735, 95% CI 0.421-1.284). In the first month of lockdown, the syphilis case-fatality ratio showed an increase of 217.3% (IRR 3.173, 95% CI 1.877-5.365); after lockdown, the number per month showed a decreasing trend of 6.7% (IRR 0.933, 95% CI 0.897-0.971); by December 2021, the syphilis case-fatality ratio had returned to the expected level (IRR 0.810, 95% CI 0.442-1.484).

#### Hepatitis B

In the first month of lockdown, there was a 17.5% (IRR 0.825, 95% CI 0.726-0.937; Figure 2D and Table 3) decrease in the number of reported hepatitis B cases; after lockdown, there was an increasing trend of 1% (IRR 1.010, 95% CI 1.002-1.018) per month; by December 2021, the number of hepatitis B cases had returned to the expected level (IRR 0.968, 95% CI 0.896-1.045).

In the first month of lockdown, there was no significant change in the number of reported hepatitis B deaths (IRR 1.038, 95% CI 0.854-1.261); after lockdown, the number showed a decreasing trend of 1.1% (IRR 0.989, 95% CI 0.979-0.999) per month; by December 2021, the number of hepatitis B deaths was significantly lower than the expected level (IRR 0.704, 95% CI 0.590-0.842). In the first month of lockdown, there was no significant change in the hepatitis B case-fatality ratio (IRR 1.312, 95% CI 0.997-1.726); after lockdown, it showed a decreasing trend of 2.4% (IRR 0.976, 95% CI 0.958-0.995); by December 2021, the hepatitis B case-fatality ratio was significantly lower than the expected level (IRR 0.709, 95% CI 0.556-0.902).

#### Hepatitis C

In the first month of lockdown, there was a 19.6% (IRR 0.804, 95% CI 0.693-0.933; Figure 2E and Table 3) decrease in the number of reported hepatitis C cases; after lockdown, there was a 1% increasing trend (IRR 1.010, 95% CI 1.001-1.019) per month; by December 2021, the number of hepatitis C cases had returned to the expected level (IRR 0.950, 95% CI 0.854-1.057).

In the first month of lockdown, there was no significant change in the number of reported hepatitis C deaths (IRR 1.006, 95% CI 0.781-1.296); after lockdown, the monthly number remained unchanged (IRR 0.996, 95% CI 0.982-1.011); by December 2021, the number of hepatitis C deaths was not significantly different from the expected level (IRR 0.926, 95% CI 0.672-1.275). In the first month of lockdown, there was a 39.4% increase (IRR 1.394, 95% CI 1.080-1.800) in the hepatitis C case-fatality ratio; after lockdown, it showed a decreasing trend of 2.1% (IRR 0.979, 95% CI 0.966-0.992) per month; by December 2021, the hepatitis C case-fatality ratio was not significantly different from the expected level (IRR 0.906, 95% CI 0.653-1.258).

### Sensitivity Analysis

Sensitivity analysis showed that the number of pairs of the Fourier term had little effect on the results (Multimedia Appendix 1, Tables S2, S3, and S4).

## Discussion

### Principal Findings

This study found that COVID-19 lockdown had a significant impact on the number of reported cases and deaths and case-fatality ratios for AIDS, gonorrhea, syphilis, hepatitis B, and hepatitis C in China. In the first month of lockdown, significant decreases were seen in reported AIDS cases (–23.4%) and deaths (–23.9%), reported gonorrhea cases (–34.3%), reported syphilis cases (–15.4%), reported hepatitis B cases (–17.5%), and reported hepatitis C cases (–19.6%). Gonorrhea, syphilis, and hepatitis C showed small increases in the number of reported deaths and case-fatality ratios in January 2020. By December 2021, nearly two years after the lockdown, the reported cases, deaths, and case-fatality ratios for each disease either reached or remained below expected levels.

Our study found that monthly reported cases of each disease showed an alarming upward trend before the COVID-19 pandemic, which was consistent with the overall trend worldwide. Since the 1980s, the number of new reported cases of STDs and BBVs has declined significantly with comprehensive prevention efforts around the world [32-34]. However, over the past decade, several countries and regions have reported an increasing trend in reported cases of STDs and BBVs. From 2009 to 2019, reported syphilis cases in Sri Lanka and reported gonorrhea and syphilis cases in Thailand showed an increasing trend [35]. A similar trend has been observed in several European countries [36]. With the widespread global epidemic of HIV, STDs and BBVs have become an increasingly serious public health problem.

In addition, the seasonality of reported cases of STDs and BBVs shown in this study is consistent with existing research. A study from Melbourne, Australia [37] found that urethral gonorrhea diagnoses among men who have sex with men (odds ratio [OR] 1.23, 95% CI 1.04-1.46) and nongonococcal urethritis diagnoses among men who have sex with women (OR 1.11, 95% CI 1.03-1.20) were higher in summer compared with winter. This may be due to increased chances of infection due to increased biological sexual desire, number of sexual partners, and sexual activity during the hot summer months [38,39], as well as increased willingness to test for STDs and BBVs in the population due to World AIDS Day (December 1). In addition, the annual seasonal pattern of monthly reported cases of the 5 infectious diseases was largely due to the annual activities of clinics and health facilities. Every year around April, health facilities in different regions in China receive their annual assignments, so most peaks in disease reporting are in the last 3 quarters. In the first quarter, there is a major holiday in China (Chinese New Year), leading to a low disease reporting level. Similar trends were observed in our study, suggesting that our data source and analytic tools were robust.

Decreases in reported AIDS, gonorrhea, syphilis, hepatitis B, and hepatitis C cases during the COVID-19 lockdown have also been observed in other countries. A national study in the United States [40] found that the numbers of weekly reported cases of chlamydia (–49.8%), gonorrhea (–71.2%), and syphilis (–63.7%) were much lower in 2020 than in week 15 of 2019. A study in Catalonia, Spain [41] found that there were 51% fewer reported cases of STDs and BBVs than expected since the beginning of the COVID-19 pandemic, reaching an average of 56% during the lockdown, with the greatest decrease of 72% for chlamydia and the least, of 22%, for syphilis. Difficulties and unwillingness to seek medical care and possible underreporting were considered as reasons for the decrease in the 5 diseases. According to a study on health service use during COVID-19 in China, all-cause visits showed a decrease of 47.6% (ranging from 24.8% in township health centers to 70% in primary care clinics) and inpatient volume showed a decrease of 47.7% (ranging from 26.8% in other health facilities to 57.8% in first-level hospitals) in February 2020 [42]. Another study, in Hong Kong, found that emergency department visits decreased by 27.4%, from 1,426,259 in 2019 to 1,035,562 in 2020 [43]. Several studies have reported that patients have delayed or avoided seeking care for fear of being infected with COVID-19 [44,45].

The decrease in the number of reported cases may also be due to NPIs such as mobility restrictions and social distancing measures affecting people’s sexual activity. A study of 967 Chinese youth [46] found that 22% of participants reported decreased sexual desire, 41% decreased frequency of sexual intercourse, 30% increased frequency of masturbation, 20% decreased alcohol consumption before or during sexual activity, and 31% deterioration in partner relationships during the pandemic and related containment measures. An online survey conducted in China [47] showed that 44% of people reported a decrease in sexual partners during COVID-19. In addition, frequent long-term lockdowns reduce interpersonal contact and public gatherings, which may also reduce the risk of transmission of STDs and BBVs in the population.

The immediate decrease in reported AIDS deaths in the first month of lockdown may be due to COVID-19 deaths among people living with AIDS being attributed to COVID-19 rather than AIDS. According to the National Health Commission of China, the cumulative number of deaths nationwide reached 4636 by the end of 2021 [48]. However, we are concerned that incorrect death certificates might obscure the truth about the deaths of HIV patients, especially during the COVID-19 pandemic, when a high work burden might have resulted in incorrect findings and documentation within hospitals and clinics. In the future, it would be valuable to elucidate the reasons behind changes in the number of deaths if more data become available. In addition, AIDS deaths were lower than the expected level 24 months after lockdown, which may be explained by the effectiveness of measures taken in China to prevent antiretroviral therapy (ART) interruption. NPIs adopted by countries or regions around the world during the COVID-19 pandemic were thought to affect the HIV care continuum for people living with HIV/AIDS [12,30,49,50]. The Chinese Center for AIDS/STD Control and Prevention released a nationwide directive on January 26, 2020, under which people living with HIV/AIDS could obtain 1 month of ART from any local HIV care clinic or hospital [51]. Designated hospitals and clinics across the country also contributed to the maintenance of ART. Hospitals in Shenzhen increased the supply of medications for a single ART collection and adopted courier delivery to ensure uninterrupted treatment for people living with HIV/AIDS during the outbreak [52]. Together, these efforts mitigated the disruption of ART to the greatest extent possible.

The increase in reported deaths or case-fatality ratios for gonorrhea, syphilis, and hepatitis C may be associated with shifts in medical resources and services to COVID-19 patients. China heavily focused the use of medical supplies, human resources, and health resources during the pandemic to respond to the explosive increase in confirmed COVID-19 cases, with some hospitals shutting down due to the risk of COVID-19 infection and some admitting only severe and critically ill COVID-19 cases [53]. Reduced willingness to seek medical care and access to care may have contributed to the disruption of treatment and the increase in deaths. However, it is important to note that the absolute numbers of deaths from gonorrhea, syphilis, and hepatitis C were very small and should not be overinterpreted.

The implementation of massive lockdowns did not change the increasing trend of reported cases of gonorrhea, hepatitis B, and hepatitis C. These short-term trends may not necessarily be stable due to the immediate impact of lockdown on STDs and BBVs in January 2020 and will need to be observed over a longer period. As China rapidly brought the domestic COVID-19 pandemic under control, the health system was able to shift resources and human resources to address other infectious diseases. Concerns about seeking health care were alleviated, with health facility visits and inpatient volume showing continued statistically significant increases after March 2020, reaching 89% and 91% of expected levels, respectively, in June 2020 [42]. Meanwhile, frequent and persistent NPIs in China such as social distancing continued to affect sexual activity and limit the spread of STDs and BBVs after the Wuhan lockdown was lifted (on April 7, 2020). By December 2021, reported cases, deaths, and case-fatality ratios for AIDS, gonorrhea, syphilis, hepatitis B, and hepatitis C either reached or remained below expected levels.

Our study used 7 years of nationwide data to estimate the impact of COVID-19 on AIDS, gonorrhea, syphilis, hepatitis B, and hepatitis C in China using 4 indicators adjusted for long-term trends and seasonality. To our knowledge, this is the first study to use interrupted time series analyses to elucidate the number of reported STD and BBV cases and deaths in China during the COVID-19 pandemic. However, our study has several limitations. First, it is an ecological study and cannot demonstrate a causal relationship between COVID-19 lockdowns and changes in trends related to AIDS, gonorrhea, syphilis, hepatitis B, and hepatitis C in China. Second, our study was based on publicly available data, which limited what kinds of data we could obtain. Due to a lack of data reported on a weekly basis, we could only define the time point for lockdown as January 2020, which may be inaccurate. Due to a lack of provincial data, we were unable to provide results for different provinces or regions in this study. Potentially, deaths due to COVID-19 and incorrect death certificates in hospitals and clinics during the COVID-19 pandemic could have affected the accuracy of this study’s results. The lack of prevalence data is also a limitation, given that the prevalence of many infectious diseases is unknown. Although the case-fatality ratio cannot be interpreted as the overall risk of death from an infection, it is the most commonly used metric because most countries collect this information [18,54,55]. As prevalence is unknown, the case-fatality ratio not only provides a crude estimate of the risk of death (such as in the COVID-19 outbreak [56]), but also allows for cross-sectional comparisons. Finally, this study was a secondary analysis of online reported data with no identifying information available to the researchers, which limits our consideration of disease intersections in the analysis. The subjects of our study should be interpreted as reported cases of a single disease rather than as individuals.

### Conclusion

In summary, these findings suggest that AIDS, gonorrhea, syphilis, hepatitis B, and hepatitis C in China were transiently affected by COVID-19 lockdown. Our findings may promote the development of STD and BBV policies and control measures in the context of the COVID-19 pandemic and the persistence of lockdowns. More long-term observations are needed to investigate trends in STDs and BBVs over time.

## Appendix

Multimedia Appendix 1 Formula for negative binominal regression models and supplementary tables.

## Abbreviations

ART: antiretroviral therapy
BBV: blood-borne virus
IRR: incidence rate ratio
NPI: nonpharmaceutical intervention
OR: odds ratio
STD: sexually transmitted disease

## Data Availability

The data sets generated during and/or analyzed during the current study are available in the public, open-access China Health and Wellness Commission repository [27].
