# Supplementary material for: The Impact of COVID-19 Lockdown on Cases of and Deaths From AIDS, Gonorrhea, Syphilis, Hepatitis B, and Hepatitis C: Interrupted Time Series Analysis
Source: JMIR Public Health Surveill. 2023 May 3;9:e40591. doi: 10.2196/40591 (PMC10193209; doi:10.2196/40591)
Supplement: Multimedia Appendix 1 [file publichealth_v9i1e40591_app1.docx]

**Appendix**

**Formula for negative binominal regression models**

*Calculating the change in outcome at NPIs, adjusted for seasonality*

$$log( Y_{t})=\beta_{0}+\beta_{1}\left( t-T \right)+\beta_{2}x+\beta_{3}x\left( t-T \right)+\beta_{4}\sin(\frac{2\pi t}{12})+\beta_{5}\cos(\frac{2\pi t}{12})+\beta_{6}\sin(\frac{4\pi t}{12})+\beta_{7}\cos(\frac{4\pi t}{12})$$

Where $Y_{t}$ is the outcome variable at time $t$, $t$ represents the elapsed time in months since the start of the study, $x$ is a dummy variable indicating the implementation of massive NPIs, $T$ is the center time, and $\sin(\frac{2\pi t}{12}), \cos(\frac{2\pi t}{12}), \sin(\frac{4\pi t}{12}), \cos(\frac{4\pi t}{12})$ are two sine/cosine pairs of Fourier terms used to adjust the seasonality.

*Counterfactual model*

$$\log Y_{t}=\beta_{0}+\beta_{1}\left( t-T \right)+\beta_{2}\sin(\frac{2\pi t}{12})+\beta_{3}\cos(\frac{2\pi t}{12})+\beta_{4}\sin(\frac{4\pi t}{12})+\beta_{5}\cos(\frac{4\pi t}{12})$$

**Supplementary Table S1 Overdispersion test of monthly AIDS, gonorrhea, syphilis, hepatitis B, and hepatitis C in China from January 2015 to December 2021**

| Overdispersion test | Statistic | *P* value |
| --- | --- | --- |
| Total |  |  |
| Cases | 185123.84 | <0.01 |
| Deaths | 9277.96 | <0.01 |
| Case-fatality ratios | 36.70 | 1 |
| AIDS |  |  |
| Cases | 29012.36 | <0.01 |
| Deaths | 9526.99 | <0.01 |
| Case-fatality ratios | 757.51 | <0.01 |
| Gonorrhea |  |  |
| Cases | 32421.96 | <0.01 |
| Deaths | 136.20 | <0.01 |
| Case-fatality ratios | 16.87 | 1 |
| Syphilis |  |  |
| Cases | 73692.53 | <0.01 |
| Deaths | 166.74 | <0.01 |
| Case-fatality ratios | 4.60 | 1 |
| Hepatitis B |  |  |
| Cases | 85215.40 | <0.01 |
| Deaths | 193.01 | <0.01 |
| Case-fatality ratios | 2.29 | 1 |
| Hepatitis C |  |  |
| Cases | 24642.10 | <0.01 |
| Deaths | 185.10 | <0.01 |
| Case-fatality ratios | 11.03 | 1 |

**Supplementary Table S2 Negative binominal segmented regression models of the impact of COVID-19 lockdown on AIDS, gonorrhea, syphilis, hepatitis B, and hepatitis C in China from January 2015 to December 2021, with 1 pairs of Fourier terms**

|  | IRR at lockdown | IRR at study end | Trend before lockdown | Trend after lockdown |
| --- | --- | --- | --- | --- |
| AIDS* |  |  |  |  |
| Cases | 0.723(0.563-0.930) | 0.726(0.589-0.895) | 1.008(1.007-1.010) | 1.009(0.992-1.026) |
| Deaths | 0.730(0.584-0.911) | 0.754(0.637-0.891) | 1.011(1.009-1.013) | 1.013(1.000-1.026) |
| Case-fatality ratios | 1.031(0.914-1.162) | 1.077(0.935-1.241) | 1.003(1.002-1.004) | 1.005(0.996-1.014) |
| Gonorrhea* | |  |  |  |
| Cases | 0.649(0.516-0.817) | 0.953(0.733-1.239) | 1.005(1.001-1.008) | 1.022(1.008-1.036) |
| Deaths | **4.436(0.538-36.598)** | 0.617(0.044-8.643) | 1.017(0.976-1.060) | 0.934(0.822-1.061) |
| Case-fatality ratios | 7.748(1.966-30.539) | 0.404(0.018-9.056) | 1.014(0.977-1.052) | 0.892(0.780-1.019) |
| Syphilis* |  |  |  |  |
| Cases | 0.836(0.749-0.933) | 0.819(0.759-0.885) | 1.005(1.004-1.007) | 1.005(0.999-1.011) |
| Deaths | 2.410(1.470-3.953) | 0.734(0.418-1.288) | 0.995(0.989-1.001) | 0.945(0.912-0.980) |
| Case-fatality ratios | 3.276(1.885-5.691) | 0.802(0.415-1.551) | 0.990(0.983-0.996) | 0.931(0.893-0.971) |
| Hepatitis B* | |  |  |  |
| Cases | 0.823(0.722-0.940) | 0.968(0.897-1.045) | 1.003(1.002-1.004) | 1.010(1.002-1.018) |
| Deaths | 1.058(0.870-1.288) | 0.702(0.588-0.838) | 1.006(1.003-1.009) | **0.988(0.978-0.998)** |
| Case-fatality ratios | 1.343(0.973-1.852) | 0.705(0.551-0.902) | 1.003(1.000-1.006) | 0.975(0.955-0.996) |
| Hepatitis C* | |  |  |  |
| Cases | 0.797(0.684-0.928) | 0.952(0.870-1.041) | 1.003(1.002-1.003) | 1.010(1.001-1.020) |
| Deaths | 1.067(0.847-1.343) | 0.911(0.666-1.246) | 0.999(0.993-1.006) | 0.992(0.980-1.005) |
| Case-fatality ratios | **1.497(1.112-2.015)** | 0.894(0.675-1.185) | 0.997(0.991-1.003) | 0.975(0.959-0.990) |

IRR= Incidence rate ratio; At lockdown: At January 2020; At study end: At December 2021; Trend: Slope change per month; Before lockdown: From January 2015 to December 2019; After lockdown: From January 2020 to December 2021; Case-fatality ratios: the number of deaths divided by the number of new cases; * Autocorrelation addressed using Newey–West standard errors to calculate CI, with lag taking the optimal value calculated.

**Supplementary Table S3 Negative binominal segmented regression models of the impact of COVID-19 lockdown on AIDS, gonorrhea, syphilis, hepatitis B, and hepatitis C in China from January 2015 to December 2021, with 3 pairs of Fourier terms**

|  | IRR at lockdown | IRR at study end | Trend before lockdown | Trend after lockdown |
| --- | --- | --- | --- | --- |
| AIDS* |  |  |  |  |
| Cases | 0.796(0.680-0.932) | 0.713(0.613-0.829) | 1.007(1.006-1.009) | 1.003(0.992-1.013) |
| Deaths | 0.783(0.693-0.885) | 0.750(0.656-0.856) | 1.011(1.009-1.012) | 1.009(1.001-1.016) |
| Case-fatality ratios | 0.990(0.899-1.091) | 1.079(0.919-1.267) | 1.003(1.002-1.004) | 1.007(0.998-1.016) |
| Gonorrhea* | |  |  |  |
| Cases | 0.662(0.530-0.827) | 0.952(0.721-1.257) | 1.004(1.001-1.008) | 1.020(1.007-1.034) |
| Deaths | **6.040(1.282-28.457)** | 0.496(0.033-7.489) | 1.015(0.980-1.052) | 0.911(0.806-1.029) |
| Case-fatality ratios | 8.538(1.886-38.659) | 0.420(0.021-8.541) | 1.013(0.976-1.050) | 0.888(0.779-1.012) |
| Syphilis* |  |  |  |  |
| Cases | 0.858(0.783-0.941) | 0.816(0.748-0.890) | 1.005(1.004-1.006) | 1.003(0.997-1.009) |
| Deaths | 2.437(1.505-3.945) | 0.734(0.412-1.310) | 0.995(0.989-1.002) | 0.945(0.912-0.978) |
| Case-fatality ratios | 3.166(1.948-5.146) | 0.811(0.446-1.477) | 0.990(0.984-0.996) | 0.933(0.901-0.967) |
| Hepatitis B* | |  |  |  |
| Cases | 0.834(0.744-0.936) | 0.966(0.902-1.034) | 1.003(1.002-1.004) | 1.009(1.003-1.016) |
| Deaths | 1.033(0.845-1.263) | 0.704(0.580-0.853) | 1.006(1.003-1.009) | **0.989(0.977-1.001)** |
| Case-fatality ratios | 1.288(0.993-1.671) | 0.711(0.570-0.887) | 1.003(1.000-1.006) | 0.977(0.961-0.994) |
| Hepatitis C* | |  |  |  |
| Cases | 0.817(0.707-0.945) | 0.947(0.866-1.037) | 1.002(1.001-1.003) | 1.009(1.000-1.018) |
| Deaths | 1.018(0.731-1.419) | 0.918(0.666-1.265) | 1.000(0.994-1.006) | 0.995(0.978-1.012) |
| Case-fatality ratios | **1.383(1.046-1.828)** | 0.908(0.646-1.275) | 0.998(0.991-1.004) | 0.979(0.963-0.996) |

IRR= Incidence rate ratio; At lockdown: At January 2020; At study end: At December 2021; Trend: Slope change per month; Before lockdown: From January 2015 to December 2019; After lockdown: From January 2020 to December 2021; Case-fatality ratios: the number of deaths divided by the number of new cases; * Autocorrelation addressed using Newey–West standard errors to calculate CI, with lag taking the optimal value calculated.

**Supplementary Table S4 Negative binominal segmented regression models of the impact of COVID-19 lockdown on AIDS, gonorrhea, syphilis, hepatitis B, and hepatitis C in China from January 2015 to December 2021, with 5 pairs of Fourier terms**

|  | IRR at lockdown | IRR at study end | Trend before lockdown | Trend after lockdown |
| --- | --- | --- | --- | --- |
| AIDS* |  |  |  |  |
| Cases | 0.822(0.709-0.954) | 0.698(0.598-0.815) | 1.007(1.006-1.008) | 1.000(0.989-1.011) |
| Deaths | 0.800(0.709-0.902) | 0.740(0.651-0.841) | 1.011(1.009-1.012) | 1.007(1.000-1.014) |
| Case-fatality ratios | 0.981(0.893-1.077) | 1.080(0.924-1.262) | 1.003(1.002-1.004) | 1.008(0.998-1.017) |
| Gonorrhea* | |  |  |  |
| Cases | 0.656(0.522-0.824) | 0.955(0.723-1.262) | 1.004(1.001-1.008) | 1.021(1.007-1.035) |
| Deaths | **4.734(1.135-19.744)** | 0.518(0.036-7.387) | 1.018(0.981-1.056) | 0.924(0.829-1.031) |
| Case-fatality ratios | 6.583(1.608-26.952) | 0.445(0.025-7.958) | 1.015(0.977-1.055) | 0.903(0.804-1.015) |
| Syphilis* |  |  |  |  |
| Cases | 0.854(0.773-0.943) | 0.817(0.752-0.888) | 1.005(1.004-1.006) | 1.003(0.997-1.010) |
| Deaths | 2.374(1.514-3.723) | 0.740(0.426-1.285) | 0.995(0.989-1.002) | 0.946(0.916-0.978) |
| Case-fatality ratios | 3.112(1.945-4.978) | 0.814(0.450-1.474) | 0.990(0.984-0.997) | 0.934(0.902-0.967) |
| Hepatitis B* | |  |  |  |
| Cases | 0.828(0.732-0.937) | 0.967(0.905-1.033) | 1.003(1.002-1.004) | 1.010(1.002-1.017) |
| Deaths | 1.028(0.855-1.236) | 0.703(0.583-0.849) | 1.006(1.003-1.009) | **0.990(0.979-1.000)** |
| Case-fatality ratios | 1.290(0.996-1.671) | 0.711(0.570-0.887) | 1.003(1.000-1.006) | 0.977(0.961-0.994) |
| Hepatitis C* | |  |  |  |
| Cases | 0.811(0.695-0.946) | 0.948(0.867-1.036) | 1.002(1.002-1.003) | 1.009(1.000-1.018) |
| Deaths | 1.000(0.711-1.407) | 0.925(0.672-1.274) | 1.000(0.994-1.006) | 0.997(0.979-1.015) |
| Case-fatality ratios | **1.355(0.985-1.863)** | 0.911(0.639-1.298) | 0.998(0.991-1.004) | 0.981(0.963-0.999) |

IRR= Incidence rate ratio; At lockdown: At January 2020; At study end: At December 2021; Trend: Slope change per month; Before lockdown: From January 2015 to December 2019; After lockdown: From January 2020 to December 2021; Case-fatality ratios: the number of deaths divided by the number of new cases; * Autocorrelation addressed using Newey–West standard errors to calculate CI, with lag taking the optimal value calculated.
